# The BH3 only Bcl-2 family member BNIP3 regulates cellular proliferation

**DOI:** 10.1371/journal.pone.0204792

**Published:** 2018-10-11

**Authors:** Amandeep Singh, Meghan Azad, Miriam D. Shymko, Elizabeth S. Henson, Sachin Katyal, David D. Eisenstat, Spencer B. Gibson

**Affiliations:** 1 Research Institute in Oncology and Hematology, CancerCare Manitoba, University of Manitoba, Winnipeg, Manitoba, Canada; 2 Department of Biochemistry and Medical Genetics, University of Manitoba, Winnipeg, Manitoba, Canada; 3 Children Hospital Research Institute of Manitoba, Winnipeg, MB, Canada; 4 Departments of Pharmacology and Therapeutics, University of Manitoba, Winnipeg, MB, Canada; 5 Departments of Oncology, Medical Genetics and Pediatrics, University of Alberta, Edmonton, Canada; Northern University, UNITED STATES

## Abstract

The BH3-only family member BNIP3 has been described as either promoting cell survival or cell death. This depends upon the level of BNIP3 expression and its cellular localization. Increased BNIP3 expression under hypoxia contributes to cell death through increased mitochondrial dysfunction. Furthermore, mice lacking BNIP3 show inhibition of ischemic cardiomyocyte apoptosis. In contrast, nuclear localization of BNIP3 contributes to blockage of apoptosis in glioma cells through repression of pro-apoptotic genes. We have discovered that mouse embryonic fibroblasts (MEFs) lacking BNIP3 expression show increased proliferation and cell number compared to wild-type cells. Furthermore, the cells lacking BNIP3 showed increased MAPK activation. Increased proliferation was not due to decreased cell death as oxidative stress induced cell death in BNIP3 null MEFs. In addition, we isolated astrocytes from wild-type or embryonic mice lacking expression of BNIP3. There was increased density and cell number in the astrocytes lacking BNIP3 expression. To confirm these results in human cells, we inducibly expressed BNIP3 in human embryonic kidney (HEK293) cells and found that induced BNIP3 reduced cell proliferation and failed to change background cell death levels. Transient over-expression of BNIP3 in the nucleus of HEK293 cells also reduced DNA synthesis. Finally, to determine whether this increased proliferation occurs in mice lacking BNIP3, we isolated brains from wild-type mice or those lacking BNIP3 expression. The mice lacking BNIP3 had increased cellularity in the brain of embryonic and adult mice. Taken together, our study describes a new function for BNIP3 in the regulation of cellular proliferation.

## Introduction

The Bcl-2 family of proteins consists of both pro-cell death and anti-cell death members divided into three distinct groups. There are the anti-apoptotic Bcl-2 family members such as Bcl-2 that when over -expressed effectively block apoptosis. Pro-cell death members such as Bax and Bak directly induce apoptosis through mitochondria dysregulation, whereas BH3-only family members regulate the degree and extent of cell death in cells [1]. BNIP3 (Bcl-2/E1B-nineteen kilodalton interacting protein) is a member of the BH3-only family consisting of four major domains: a PEST domain that causes BNIP3 degradation, a BH3 domain (Bcl-2 homology 3) that characterizes BNIP3 as a member of the BH3-only Bcl-2 family, a CD (conserved domain) that is conserved from *C*. *elegans* to humans, and a TM (transmembrane domain), which integrates BNIP3 into the outer membrane of the mitochondria [2]. Deletions of the CD or BH3 domains fail to affect cell death but deletion of the TM domain blocks BNIP3 induced cell death, due to lack of BNIP3 localization to the mitochondria [3].

BNIP3 function has been associated with both cell death and cell survival. BNIP3 is up-regulated following hypoxia through the activation of hypoxia inducing factor 1 (HIF1) and induces cell death by opening the permeability transition (PT) pore, leading to loss of mitochondrial membrane potential and reactive oxygen species (ROS) production at the mitochondria [2]. BNIP3 has also been involved in autophagic cell death in malignant gliomas in response to hypoxia, ceramide or arsenic trioxide treatment [4]. In contrast, BNIP3 has been associated with cell survival. Over-expression of BNIP3 induces mitophagy (selective degradation of mitochondria) contributing to cell survival. In solid tumors, BNIP3 is expressed at high levels in viable cells within hypoxic regions suggesting it could contribute to cell survival [5]. We previously found that BNIP3 is localized to the nucleus in the majority of glioblastoma (GBM) tumors and in a subset of normal astrocytes. Nuclear BNIP3 associates with a complex consisting of PTB-associating splicing factor (PSF) and histone deacetylase 1 (HDAC1) contributing to transcriptional repression of the apoptosis inducing factor (AIF) gene. Reduction in AIF expression decreased temozolomide sensitivity and TRAIL-induced apoptosis in glioma cells. Furthermore, nuclear BNIP3 expression in GBM tumors correlates with decreased AIF expression [6]. This indicates that nuclear BNIP3 contributes to cell survival through repression of pro-cell death genes. Other BNIP3 functions have not been described.

Dorn and colleagues have shown through BNIP3 knockout mice that BNIP3 is a major determinant of post-ischemic apoptosis in the heart, since these mice demonstrate reduced apoptosis in the myocardium after surgical ischemic/reperfusion injury [7]. Furthermore, forced cardiac expression of BNIP3 increases cardiomyocyte apoptosis in unstressed mice [8]. The livers of BNIP3 knockout mice also exhibit reduced lipid metabolism, increased mitochondrial mass, increased reactive oxygen species and show features of steatohepatitis [9]. Otherwise, these mice have normal life span and appear to be healthy [7]. Currently, little is known about the role of BNIP3 in normal function and development. In this study, we investigated the effect of BNIP3 on cellular proliferation in mouse embryonic fibroblasts, astrocytes and in the human cell line HEK293. In addition, we assessed cell numbers in the brains of BNIP3 knockout mice compared to littermate controls.

## Material and methods

### Cell culture and mouse model

MEF cells (obtained from Animal Care Services, University of Manitoba) and HEK293 cells (obtained from ATCC) were cultured in DMEM media (Thermofisher) consisting of 4mM L-glutamine, 4mM sodium pyruvate, 4.5 microg/mL glucose and supplemented with either 10% fetal bovine serum (MEFs) or 5% fetal bovine serum (HEK293), and 100 units/ml penicillin/streptomycin. The MEFs were also supplied with 0.1 mM beta-mercaptoethanol in culture to help reduce toxic oxygen radicals. The cultures were maintained in a humidified incubator with 5% CO_2_ at 37°C. For hypoxia treatment of MEFs to overexpress BNIP3, cultures were maintained in a hypoxic chamber (Forma Scientific, Waltham, MA) at 37°C with <1% O_2_ and 5% CO_2_ balanced with N_2_. Primary mouse astrocytes were cultured in custom astrocyte-selective media: CaCl2 2H2O (0.256 g/L), MgSO4 (0.96 g/L), KCl (0.402 g/L), NaCl (6.78 g/L), NaH2PO4 (0.12 g/L), L-Glutamine (0.294 g/L), Phenol Red (0.02 g/L), D-Glucose (1.352 g/L), NaHCO3 (2.2 g/L) (all from Sigma-Aldrich, Oakville ON); with the following liquid supplements from Invitrogen (Burlington, ON): MEM Amino Acids (4% v/v; Invitrogen), MEM Vitamins (4% v/v; Invitrogen), 33 units/mL penicillin and 33 μg/mL streptomycin. After isolation in serum-free media, astrocytes were supplemented with 10% or 7% FBS. Vigorous swirling was used to detach non-astrocytic cells at the time of passaging and/or changing media.

The BNIP3-null mouse model was kindly provided by Dr. Gerald Dorn (Washington University School of Medicine). The BNIP3-null allele was generated by replacing exons 2 and 3 of the BNIP3 gene with a neomycin resistance cassette through homologous recombination [7]. This insertion truncates the BNIP3 protein prior to its critical BH3 and transmembrane domains. The BNIP3-null allele was validated by Dorn and colleagues, who showed by Northern blot and immunoblot assays that neither BNIP3 RNA nor BNIP3 protein were detectable in homozygous BNIP3-null mice. Unless otherwise specified, heterozygotes were crossed to generate all wild type and BNIP3-null mice studied.

The adult mice were sacrificed by cervical dislocation after anesthesia with chloral hydrate.

In addition, animals are housed in autoclavable polycarbonate shoe box cages provided with aspen bedding chips which allows for group housing a maximum of 4 adult mice. Environmental enrichment is provided in the form of enviro-dri paper to enhance nest building in addition to paper or polycarbonate shepherd shacks. Diet consists of LabDiet RMH 3000 which assures minimal biological variation in long term studies. Standard chlorinated water is provided in autoclavable glass bottles. Clean cages, fresh aspen bedding, enviro-dri, and fresh water is provided on a weekly basis.

### RT-PCR

Four MEF cell lines were used for the proliferation experiments- two from wild-type mice (WT1, WT2) and two from BNIP3 knockout mice (KO1, KO2). The cell lines were validated for presence or absence of BNIP3 expression using a real time reverse transcriptase polymerase chain reaction (qRT-PCR) assay. RNA was isolated from cells using the RNeasy plus mini kit (Qiagen) following the manufacturer’s protocol. 1μg of RNA was used to synthesize cDNA using qScript cDNA supermix (Quantabio) according to the manufacturer’s protocol. The BNIP3 primers and iTaq Universal sybr green supermix were purchased from Bio-Rad and the reaction was run on Bio-rad CFX96 thermal cycler according to the manufacturer’s protocol. GAPDH and Brachyury were used as reference genes.

### Western blot

Cells were lysed for total protein in 1% NP40, 2mM EDTA, 10% glycerol, 20mM Tris, 150 mM NaCl, 1 protease inhibitor tablet/10mL (Sigma) and 1μL/mL phosphatase inhibitor cocktail (Thermofisher) lysis buffer. The lysates were separated by SDS-PAGE and transferred to 0.45 microm nitrocellulose membranes. The membranes were blocked with 5% skim milk in PBST and were incubated with antibodies against BNIP3 (Abcam), p-MAPK (Cell Signaling), MAPK (1:1000, Cell Signaling), LC3 (Abcam), and GAPDH (Millipore Sigma). The western blots were visualized using ImageQuant LAS 500.

### Edu cell proliferation assay

MEF cells were serum starved overnight at ~80% confluence to synchronize cell cycles. Cells were then harvested and 1x10^6^ cells were plated in replicates of three in 10-cm dishes. Cells were cultured under normal conditions. After 1 hour, Edu (5-ethynyl-2'-deoxyuridine) was added in a final concentration of 10μM, and cells were further incubated for 2 hours. The appropriate incubation times were determined through an initial time course experiment. Edu is a thymidine analog, and is incorporated into DNA of proliferating cells. After incubation, cells were harvested and stained for Edu with a covalently binding azide dye according to the manufacturer’s protocol. The cells were counted using a Novocyte flow cytometer (50,000 events recorded per sample).

### Real time cellular analysis

The RTCA instrument utilizes special 16-well plates coated with electrodes at the bottom. These plates are placed on an instrument that transmits electrical impulses from one end of the plate to the other. In the absence of cells, there is a minimal loss of electrical impulse from one end to the other. Once cells are seeded in the plates, the electrical signal is impeded by the cells and this loss of signal is proportional to the number of cells in a well and a cell index is derived by the accompanying software. The instrument was first blanked using culture media. 5000 cells were seeded per well in replicates of three wells per cell line. Appropriate seeding number was determined by running RTCA for different cell counts ranging from 2000 to 10,000 cells per well. The cells were left undisturbed in the incubator and electrical impulses were measured every 15 minutes for six days.

### Cytation V cell count assay

This assay was done in 6-well dishes. 10,000 cells (determined by an initial cell density experiment) were seeded on Day 0 per well for both wild-type and BNIP3 knockout MEF cell lines (3 wells/cell line per day; 18 total wells/cell line for six days), and cultured normally. Each day, three wells for each cell line were stained with NucBlue Live cell staining reagent which stains the nucleus of live cells. The wells were imaged using a Cytation V instrument, and four representative images were taken per well. The cells in the images were counted using Gen 5 software, which accompanies the Cytation V system. The software analyzes images, outlines the positively stained blue nuclei and provides the cell count in the image. This was repeated over six days to obtain a cell growth curve.

### Transfection experiments

HEK293 cells were plated two days before transfection to achieve ~70–80% confluence. Cells were transfected using Lipofectamine 3000 (Thermofisher) according to the manufacturer’s instructions. Cells were transfected with 5 microgram of either pcDNA3.1 (control) vector or pcDNA3.1-NLS-BNIP3 (nuclear targeted BNIP3 expression vector). The cells were incubated normally for 24 hours. At 24 hours, cells were serum starved overnight to synchronize cell cycle. Next day, transfected cells were harvested and (a) 1x10^6^ cells were seeded back into 10-cm dishes for Edu cell proliferation assay, whereas (b) remaining cells were lysed to extract protein for western blot analysis.

### Constructs for TetON inducible expression system

An inducible tetracycline-regulated BNIP3 expression system was developed using Invitrogen’s T- Rex System. The T-Rex system consists of two components: the pcDNA6/TR regulatory vector encodes the tetracycline repressor (TR) protein, and the pcDNA4/TO inducible expression vector contains the gene of interest (GOI) controlled by the cytomegalovirus (CMV) promoter and two upstream tetracycline operator sequences (TetO2). The BNIP3 gene had previously been subcloned into the pcDNA4/TO inducible expression vector (Dr. Adrian Harris, Weatherall Institute of Molecular Medicine). We established stable HEK293 pcDNA6/TR-expressing cell lines. Transfections were performed as described above. Stably-transfected cells were selected with 10 microgram/mL blasticidin (Invitrogen). Transfected cells were treated with or without 1microg/mL doxycycline (Sigma-Aldrich) and incubated for 24 hours to induce beta-gal expression. Then, cells were assayed for beta-gal activity using the beta-gal Enzyme Assay System (Promega) according to the manufacturer’s instructions. Inducible expression was determined using the beta-gal Enzyme Assay System or by anti-BNIP3 Western Blot.

### GFP-LC3 localization

LC3 is recruited to the autophagosome membrane during autophagy. Cells were transiently transfected with the GFP-LC3 expression vector. Cells were examined under an Olympus BX51 fluorescent microscope to assess GFP-LC3 localization. GFP-LC3 presents a diffuse distribution under control conditions, whereas a punctate pattern of GFP-LC3 expression is indicative of autophagy. The GFP-LC3 expression vector was kindly provided by Drs. N. Mizushima and T. Yoshimori (Tokyo Medical and Dental University).

### MTT assay for cell viability

Equal numbers of wild type and BNIP3 wild-type and knockout astrocytes were plated in 96- well dishes on “day 0” (increasing cell densities from 5,000 to 50,000 cells per well, in triplicate). The MTT assay was performed on days 1, 2, 4, 7 and 10 to measure cell proliferation. Briefly, cells were incubated in fresh media containing MTT reagent (0.2 mg/mL) for 1 hour at 37 Celsius to permit metabolism of MTT to formazan in viable cells. After this incubation, media was aspirated and replaced with 125 microL DMSO per well, to dissolve the purple formazan crystals. Absorbance (which is proportional to the number of viable cells in each well) was measured at 540 nm on a microplate reader.

### Immunofluorescence

Cultured cells were either grown on coverslips, or harvested and adhered to glass slides Cells on slides or coverslips were fixed in 3.7% formaldehyde in PBS for 15 minutes at room temperature, and stored in fixing solution at 4 Celsius. Following three washes with PBS (0.1% NP40), slides/coverslips were incubated with primary antibody in PBS (0.1% NP40, 10% FBS) and secondary antibody for 1 hour at room temperature. After three final washes, slides/coverslips were mounted with DAPI + antifade reagent (BioRad) to counterstain for nuclei. Fluorescence was visualized and captured using a Zeiss Axiophot microscope.

FFPE mouse whole were baked in an oven (70 Celsius) for 20 min. The slides were deparaffinized, rehydrated and washed with H_2_O for 5 min. Antigen presentation was completed by incubating the slides in a pressure cooker for 20 min filled with citrate buffer (10 mM citric acid monohydrate, pH to 6.0). The slides were removed, cooled to room temperature (RT), and then washed three times for 5 min in PBS-T (0.5% Triton X100). Blocking solution (1X PBS, 0.2% Triton X100, 0.02% sodium azide, 5% goat serum and 0.1% bovine serum albumin) was added to each slide for 2 hrs at RT. Primary antibodies (anti-Ki-67 [#12202P] at 1:100, and anti-cleaved caspase 3 [#9661] at 1:100 dilution (Cell Signaling)) were diluted in blocking solution and added to slides. The slides were incubated at 4 Celsius overnight and subsequently washed. The appropriate biotinylated secondary antibody (1:200 dilution, Vector Inc.) was prepared in blocking solution and added to the slides for 2 hrs at RT then washed three times. Strepavidin conjugated to Oregon Green (Molecular Probes) was diluted in blocking solution (15 microgram/ml) then added to slides and incubated for 2 hrs at RT in the dark. Slow Fade Gold with Dapi (Invitrogen) was added to each slide and a coverslip was placed over the brain sections. Fluorescence was visualized and captured using an Olympus BX51 fluorescent microscope with a Photometrics Cool Snap CF camera. No primary control slides with only secondary and tertiary antibodies added were used to measure non-specific auto-fluorescence.

### Whole brain paraffin-embedding, sectioning and H&E staining

Paraffin-embedding and sectioning of perfusion-fixed whole brains was performed as a contract service by the Manitoba Breast Tumor Bank. Every 50 micrometer, two 10 micrometer horizontal sections were retained and mounted on separate glass slides; one was processed for hematoxylin and eosin (H&E) staining by the Tumor Bank, while the other was stored for future use. Matched stained and unstained sections were stored together in plastic slide boxes at room temperature. Images of H&E-stained sections were captured on an Olympus BX51 microscope equipped with the Olympus DP70 Digital Camera System and used to compare gross brain morphology in matched wild-type and BNIP3-null specimens. Anatomical features were identified using the online High Resolution Mouse Brain Atlas (www.hms.harvard.edu/research/brain/atlas.html). Cellularity in anatomically-matched sections was determined by analyzing 20x and 40x images with the “counter” function in Image Pro Plus software. At least eight different areas (selected to cover a variety of anatomical brain regions) were analyzed and compared in each brain. Three pairs of adult wildtype and BNIP3 knockout littermates were compared, in addition to four E18.5 embryos (2 wild-type and 2 BNIP3 knockout) from a single heterozygous cross. This research was approved by the Bannatyne Campus Animal Care Committee, University of Manitoba. Animal Care and Veterinary Services, Research Ethics and Compliance, Bannatyne Campus Animal Care Committee, Approval number 15–074

### Isolation of primary astrocytes from neonatal mice

To harvest astrocytes from neonates (under 96 hours old) resulting from homozygous wild-type or BNIP3 null crosses. Neonates are removed from the facility live in a clean cage with bedding material and a small heating pad warmed to body temperature and wrapped in green cloths. Neonates are brought to a flow hood which is designated for the isolation and maintenance of primary cell cultures exclusively. Neonates are sacrificed immediately by decapitation with scissors. Whole brains were immediately removed and placed in ice-cold serum-free astrocyte culture medium. The cortices were transferred to a fresh dish of ice-cold serum-free culture media. Cortices from each litter were combined and chopped into small pieces (<1mm3) and then transferred to a 50 mL conical centrifuge tube with 15 mL of serum-free culture media. The tissue was mechanically dissociated by vortexing to achieve a single-cell suspension. Cells were seeded in astrocyte culture media with 10% FBS at a density of 6x105 cells/mL. “Mature” astrocyte cultures were tested for purity by immunofluorescence staining for the astrocyte-specific marker protein GFAP (glial fibrillary acidic protein). Primary mouse astrocytes were cultured in custom astrocyte-selective media as described above. After isolation in serum-free media, astrocytes were supplemented with 10% FBS. Vigorous swirling was used to detach non-astrocytic cells at the time of passaging and/or changing media.

### Statistical analysis

The two wildtype and two knockout samples were compared using One-way ANOVA followed by Tukey’s post hoc test in Graphpad Prism (WT1 vs KO1; WT1 vs KO2; WT2 vs KO1 and WT2 vs KO2). The highest p-value from post hoc test is reported for all wildtype and knockout comparisons.

## Results

### Mouse embryonic fibroblasts and astrocytes lacking BNIP3 expression show increased proliferation

**Fig 1:**
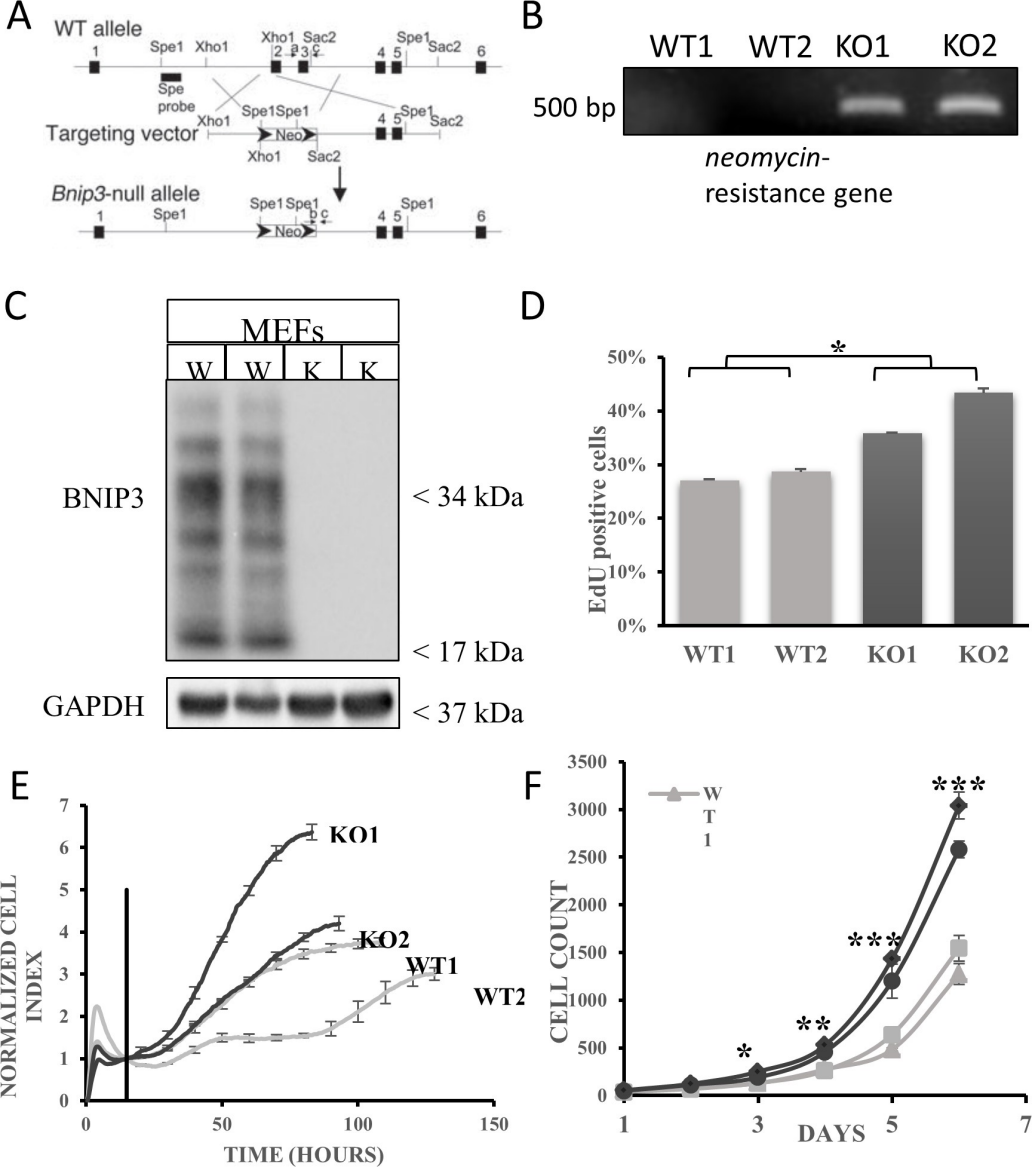
MEF cells lacking BNIP3 have increased proliferation compared with MEF cells expressing BNIP3. A) Bnip3-null allele was created by replacing exons 2 and 3 with a neomycin resistance cassette. (B) Reverse transcription polymerase chain reaction (RT-PCR) was performed on total RNA isolated from MEFs using primers against neomycin-resistance gene. The PCR products were run on a 1% agarose gel at 80V for 1.5 hours and visualized under UV light with Chemidoc XRS+ instrument (Bio-Rad). A single band at ~500bp is observed (expected size is 492 bp). (C) Total lysates from MEFs were western blotted for BNIP3. Cells were incubated under hypoxia for 24 hours before lysing to over-express BNIP3. The blot was stripped and reprobed with GAPDH as loading control. (D) The Click iT EdU cell proliferation assay was performed and Edu positive cells were counted using a flow cytometer. The results show Edu positive cells as a percentage of total cells in the sample, and are an average of three replicates. The error bars show standard error of the mean. Tukey’s post hoc test was performed to measure statistical significance (***p<0.0001). (E) Cell counts were monitored using an RTCA instrument over 5 days. The results shown are representative of three independent experiments. The error bars show standard deviation. (F) Cells were incubated under normal conditions and counted with a Cytation V instrument over 6 days. The cell count shown is an average of three independent wells (4 measurements per well). The error bars show standard error of the mean. Tukey’s post hoc test was used to calculate statistical significance (* p<0.05, ** p<0.02, *** p<0.005). Results shown are representative of three independent experiments.

Mouse embryonic fibroblast (MEF) cells were cultured and grown in tissue culture condition from wild-type and BNIP3 knockout neonates. The wild-type and BNIP3 knockout MEFs were verified with qRT-PCR and western blot for knockout of BNIP3 expression based on the genotype of the BNIP3 gene (Fig 1A). In Fig 1B, wild-type MEFs have a much higher expression of BNIP3 compared to BNIP3 knockout MEFs at the mRNA level and confirmed by western blot (Fig 1C and S1 Fig) following incubation under hypoxia for 24 hours. The multiple bands for BNIP3 represents degradation products under hypoxia.

**Fig 2:**
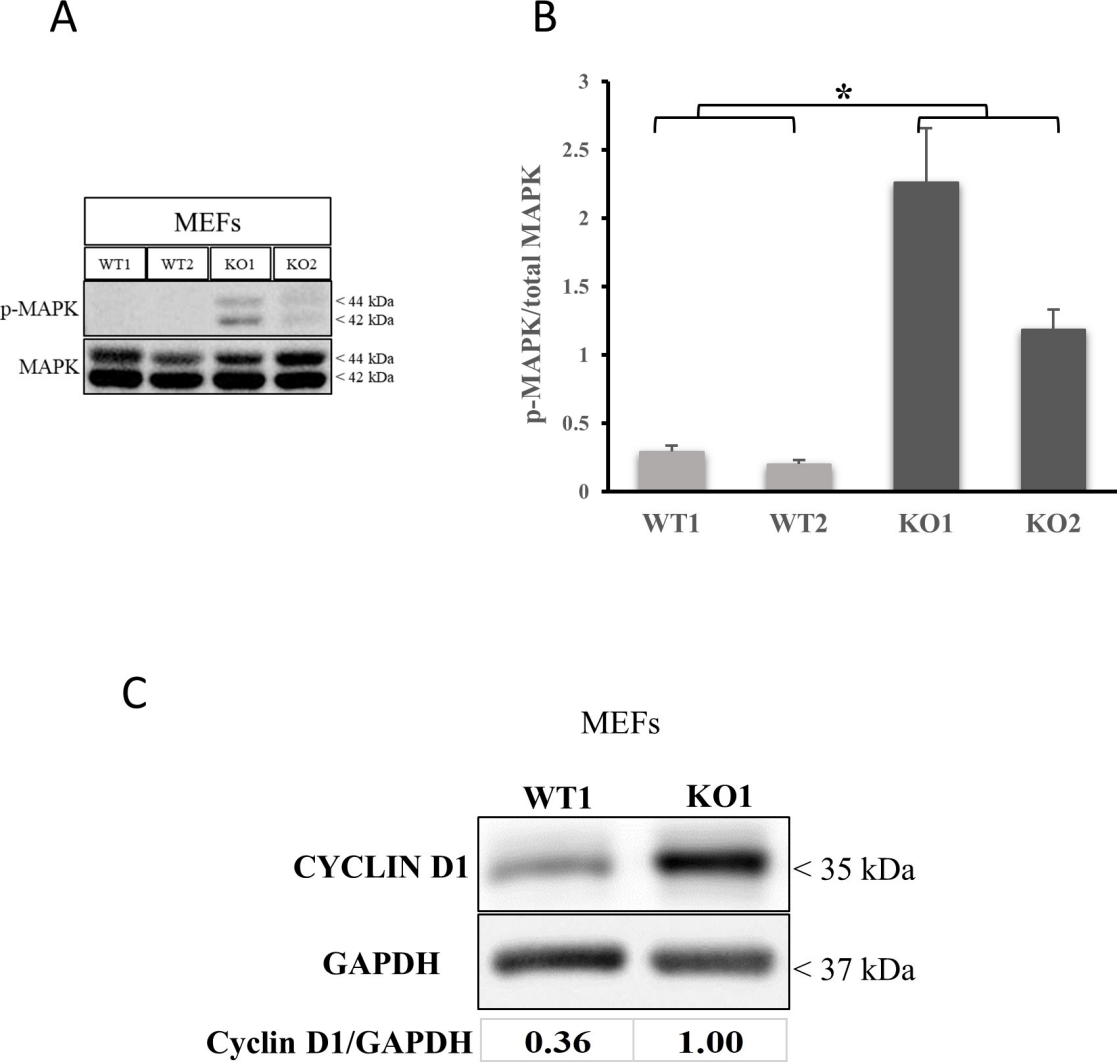
MEF cells lacking BNIP3 show higher levels of MAPK activation compared to MEF cells expressing BNIP3. (A) Total lysates from wild-type and BNIP3 knockout MEFs were western blotted for phospho-MAPK. The blot was stripped and reprobed with total MAPK and GAPDH as loading controls. (B) The protein levels of p-MAPK and total MAPK were measured using ImageJ software. The ratio of p-MAPK/total MAPK was plotted to compare the activation of MAPK. The results are an average of three replicates. The error bars show standard error of the mean. Tukey’s post hoc test was used to calculate statistical significance (*p<0.05). Results shown are representative of three independent experiments Total lysates from wild-type and BNIP3 knockout MEFs were western blotted for Cyclin D1. The blot was stripped and reprobed with GAPDH as loading control. The protein levels were quantified using ImageJ software. The ratio of Cyclin D1 to GAPDH was calculated and normalized to the highest level.

We then analyzed cell proliferation by measuring the rate of EdU incorporation into the nuclei of proliferating cells. The Edu cell proliferation assay showed that MEF cells lacking BNIP3 expression have a higher percentage of cells in the DNA synthesis phase of the cell cycle compared to wild-type similar to astrocytes (Fig 1D). In addition we used the real time cell assay (RCTA) to measure the rate of proliferation over a 5 day time course. This assay revealed that MEF cells lacking BNIP3 expression proliferated at a higher rate than the MEF cells expressing wild-type BNIP3 (Fig 1E). This was confirmed by counting cell numbers of a similar time course using the Cytation V imaging station (Fig 1F). The cell number of MEF cells lacking BNIP3 increased faster over 5 days compared to MEF expressing wild-type BNIP3. The MEF cells were then lysed and western blotted for MAPK activation by western blotting. MEF cells lacking BNIP3 had elevated levels of MAPK activation compared to MEF cells expressing wild-type BNIP3 (Fig 2). This was further confirmed by cyclin D1 expression (Fig 2C). When MEF cells lacking BNIP3 were synchronized with the cell cycle, the amount of cyclin D1 decreased compared to wild type indicating the BNIP3 lacking MEF cells were entering the cell cycle faster than wild type MEF cells (S2 Fig). This result indicates that MEF cells lacking BNIP3 have increased cellular proliferation.

**Fig 3:**
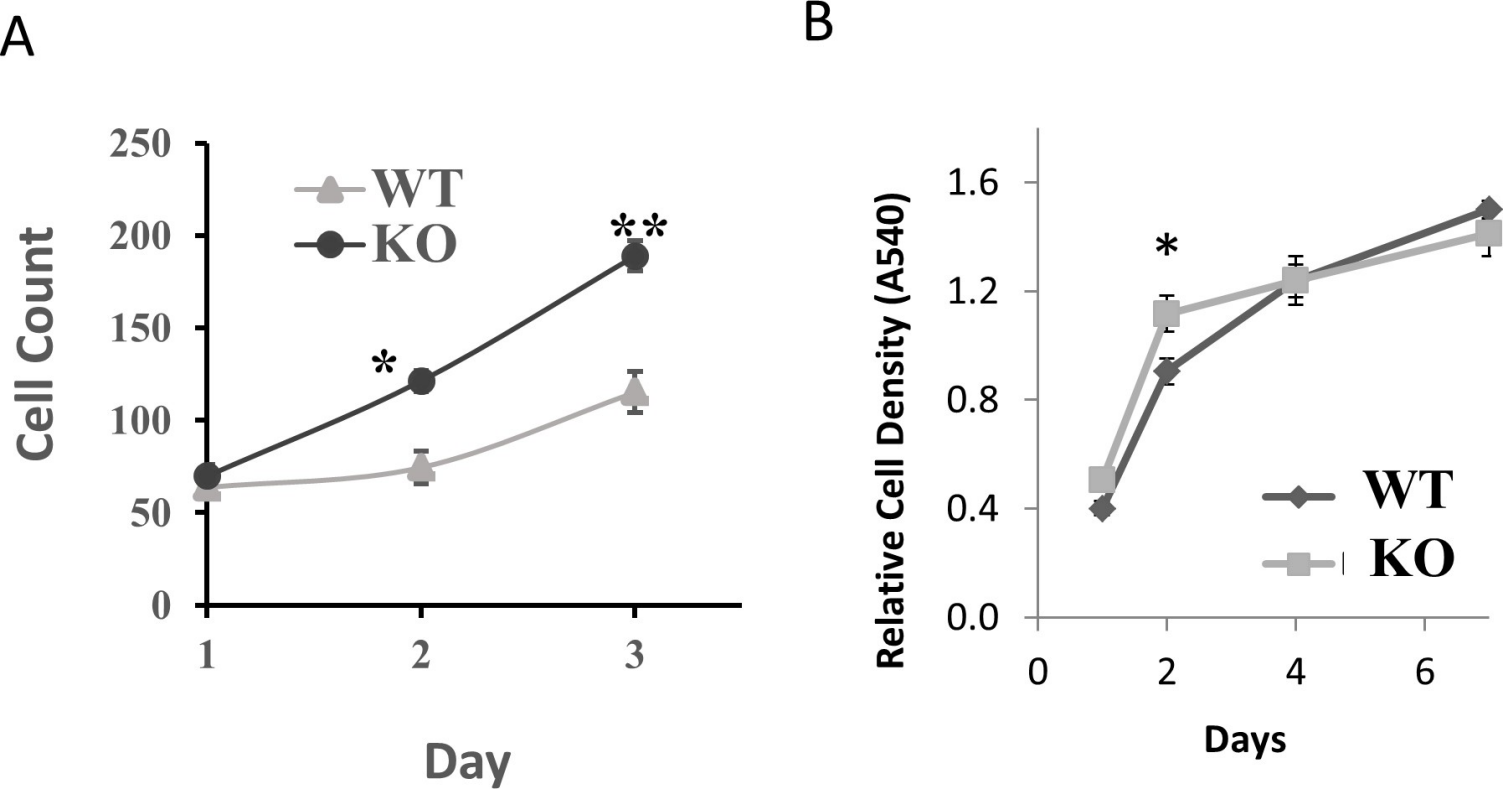
Loss of BNIP3 increased proliferation in cultured primary astrocytes. (A) Equivalent quantities of wild-type (WT) and BNIP3 knockout (KO) astrocytes isolated from neonatal brains of mice were seeded in 6-well plates, and plates were analyzed using the Cytation V instrument. The cell count shown is an average of three independent wells. (B) Equivalent numbers of wild type (WT) and BNIP3 knockout (KO) astrocytes were plated in triplicate in 96-well plates and allowed to replicate for 1 to 6 days. Relative cell density was measured by quantifying the amount of MTT reagent metabolized to purple formazan in each well by colorimetric assay. The error bars show standard error of the mean. Student’s t-test was used to calculate statistical significance. (ns: not significant, *: p<0.05, **: p<0.01).

To confirm these results in another cell type, we isolated astrocytes from the brains of neonate mice as described in the Materials and Methods section. We then analyzed cell number by Cytation V and cell density by MTT assay, and found increases in cell number after three days (Fig 3A) and increase in cell density after two days (Fig 3B) in astrocytes lacking BNIP3 expression compared to wild-type astrocytes. After four days, both astrocytes were similar in cell density (Fig 3B). Together, these results indicate that cells lacking BNIP3 have increased proliferative capacity.

### MEF cells lacking BNIP3 expression failed to show differences in cell death

**Fig 4:**
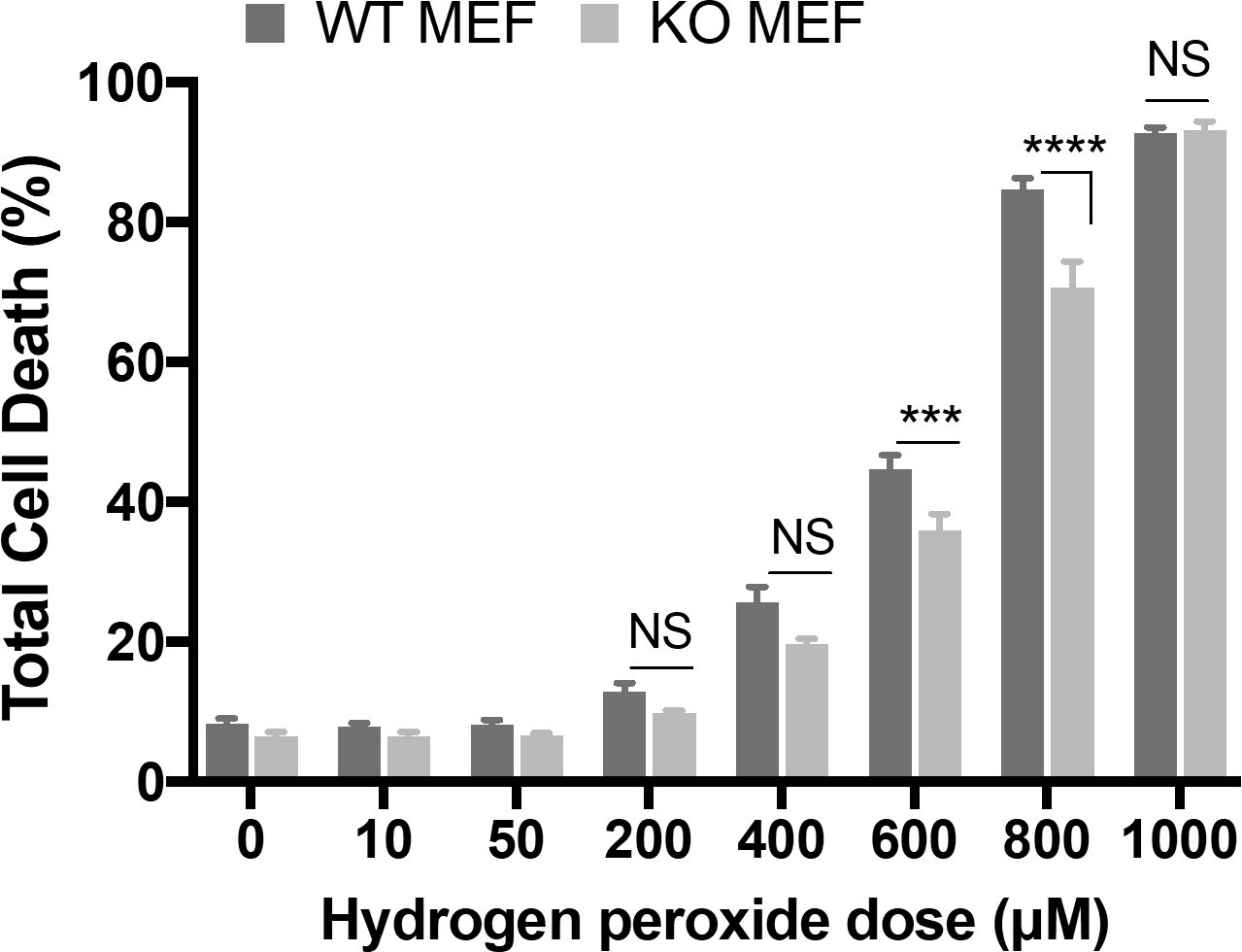
MEF cells lacking BNIP3 show no differences in background cell death and only a slight reduction in oxidative stress-induced cell death. Mean cell death of BNIP3 wild-type (WT) and knockout (KO) Murine Embryonic Fibroblast (MEF) cells treated with hydrogen peroxide over a range of concentrations (10–1000 microM) for 24 hours. Total cell death was measured by flow cytometry with trypan blue staining. Bars represent standard error of three independent experiments.

We investigated whether cells lacking BNIP3 expression have altered cell death responses. MEF cells were treated with a range of hydrogen peroxide concentrations for 24 hours and then total cell death was measured by flow cytometry with trypan blue staining (Fig 4). Cell death was only moderately lower in MEF cells lacking BNIP3 than in wild-type MEF cells when treated with 200–800 microM hydrogen peroxide. Background cell death also remained unchanged between MEF cells lacking BNIP3 or expressing wild-type BNIP3. This indicates that lack of BNIP3 fails to significantly alter cell death induced by hydrogen peroxide in MEF cells.

### Over expression of BNIP3 reduces cell proliferation in HEK 293 cells

**Fig 5:**
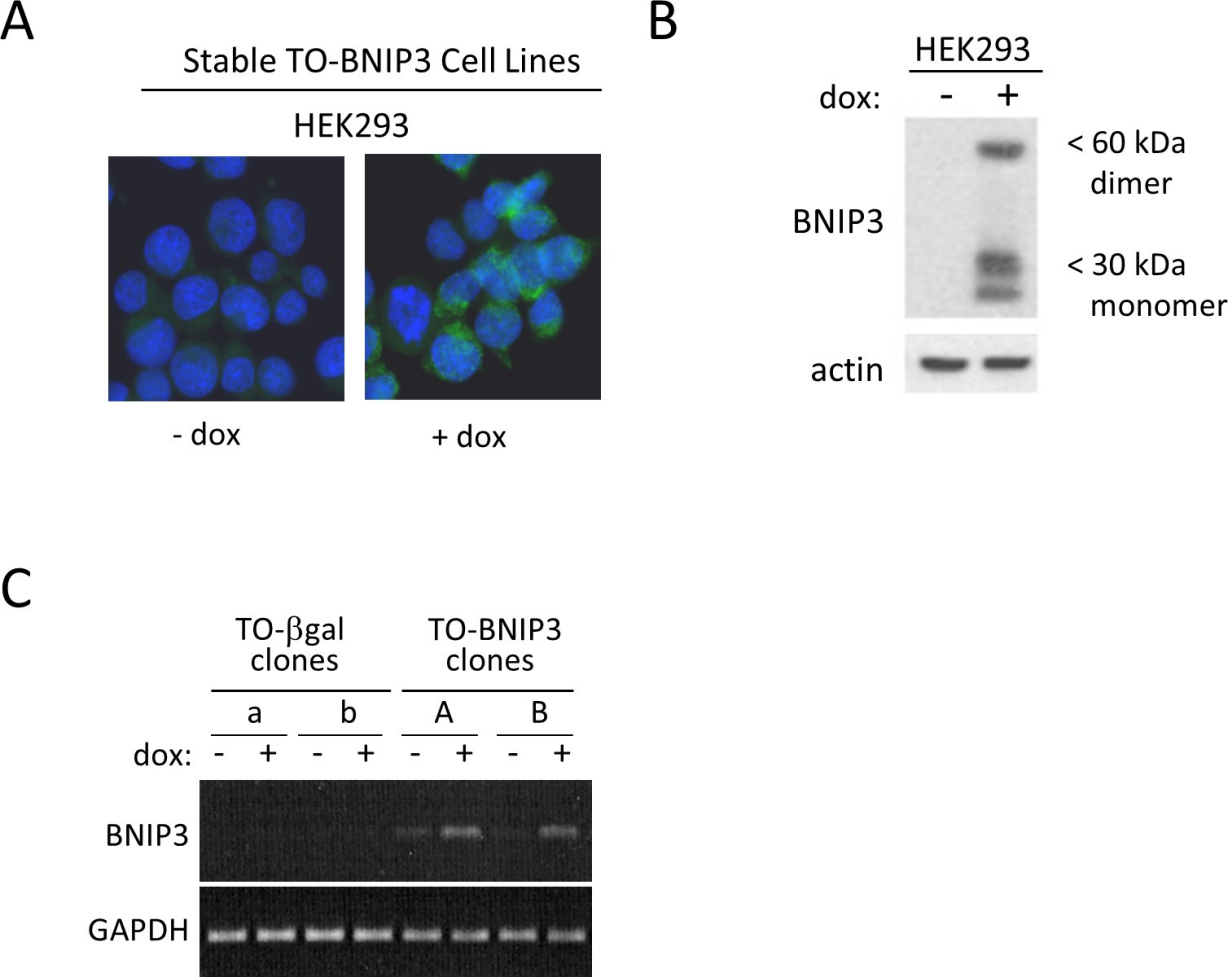
Stable deoxycycline inducible expression of BNIP3. HEK293 cells were engineered to stably co-express the Tet Repressor protein (TR) and tetracycline-inducible BNIP3 (TO-BNIP3) or beta-galactosidase (TO-β-gal) as described in the Materials & Methods. Multiple clones were established for each cell line. Addition of the tetracycline analog, doxycycline (dox), for 24 hours relieves TR repression and induces BNIP3 expression as detected by immunofluorescence (A), Western blot (B) and RT-PCR. In (A), nuclei are visualized by Hoescht dye (blue) and BNIP3 is seen in the cytoplasm (green fluorescence).

**Fig 6:**
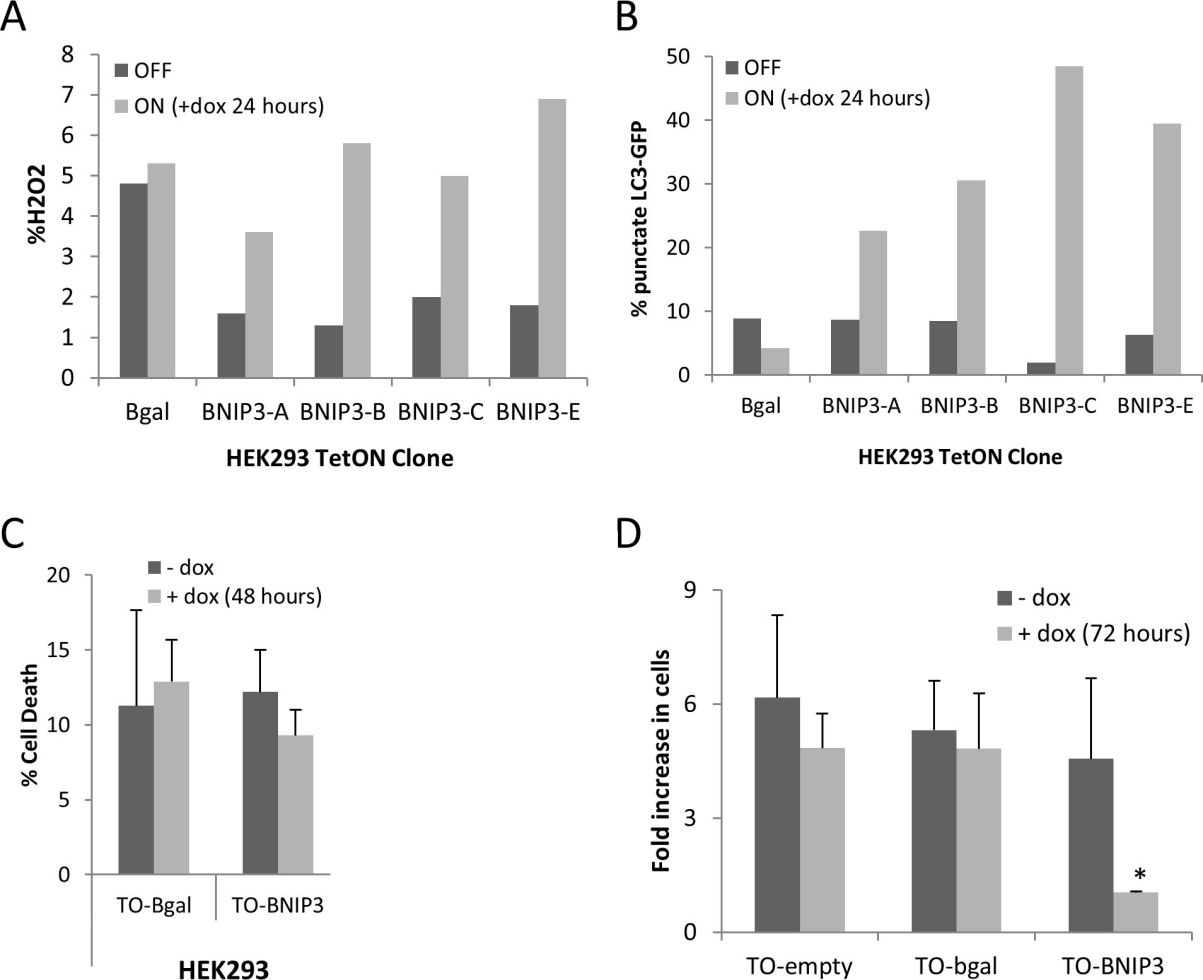
BNIP3 induction in HEK 293 cells reduces cell growth. HEK293 cells were engineered to stably co-express the Tet Repressor protein (TR) and tetracycline-inducible BNIP3 (TO-BNIP3) or beta-galactosidase (TO-β-gal) as described in the Materials & Methods. Cells were untreated or treated with doxycycline (1 microg/mL) to induce gene expression. (A) Reactive oxygen species (ROS) production was increased upon BNIP3 (but not β-gal) induction. ROS were detected using the dye CM-H2DCFDA, which is oxidized to green fluorescent DCF (dichlorofluorescein) by hydrogen peroxide. (B) Autophagy was also increased upon BNIP3 (but not beta-gal) induction, as determined by GFP-LC3 localization. TO-cells were transiently transfected with GFP-LC3. 24 hours after transfection, cells were untreated or treated with doxycycline for an additional 24 hours. (C) Cell death was not induced upon BNIP3 or β-gal induction, as determined by the membrane permeability assay. (D) Cell growth was severely restricted upon BNIP3 (but not beta-gal) induction in HEK293 cells. For each cell type, 1.5x105 cells were seeded in one well of a 6-well culture dish, with or without doxycycline (gene induction). Three days later, the number of adherent cells in each well was determined using a Beckman Coulter Counter. The fold-increase represents the ratio of cells on day three compared to the number of cells originally seeded. Results represent the average of three independent experiments; error bars represent standard deviation. *p < .01.

Using Invitrogen’s TREx System, we established stable HEK293 cell lines with tetracycline-regulated BNIP3. In these “TetON” cells (TO-BNIP3 cells), BNIP3 expression is highly induced by doxycycline (a stable analogue of tetracycline), as detected by immunofluorescence, Western blot and RT-PCR (Fig 5A–5C). We and others have shown that BNIP3 triggers mitochondrial ROS production and autophagy [10]. As expected, induced BNIP3 expression in TO-BNIP3 cells led to increased ROS production and autophagy, which was not observed following beta-gal induction (Fig 6A and 6B). BNIP3 induction, however, did not result in cell death, even after 48 hours. Cell death remained unchanged at 9–12% regardless of the level of BNIP3 induction, which was comparable to TO- beta gal cells with or without induction (6–13%) (Fig 6C). Although cell death was not induced upon BNIP3 expression, proliferation was reduced (Fig 6D). Three days after seeding, the number of TO- β-gal cells had increased 5.3-fold, which was unchanged (4.8-fold) in the presence of doxycycline. Proliferation of TO-BNIP3 cells was comparable in the absence of doxycycline (4.6-fold increase over three days); however, doxycycline treatment (i.e. BNIP3 induction) at the time of seeding restricted proliferation almost completely (1.05- fold increase). Thus, induction of BNIP3 expression reduced cell growth independent of cell death.

**Fig 7:**
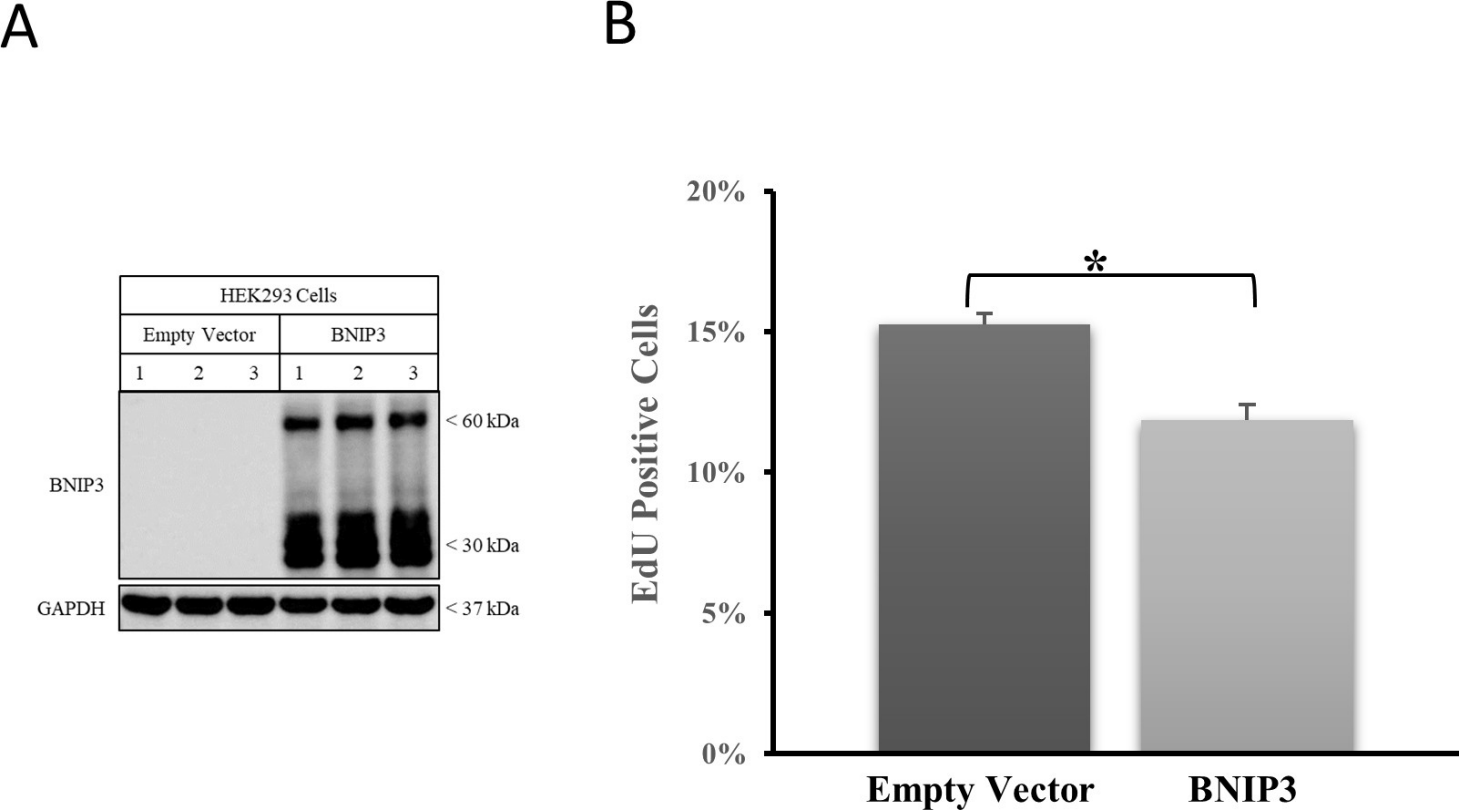
HEK293 cells over-expressing nuclear BNIP3 have lower proliferation than cells transfected with empty control vector. (A) HEK293 cells were transfected with either an empty control vector or nuclear BNIP3 expression vector. Total lysates from transfected cells were western blotted for BNIP3. The blot was stripped and reprobed with GAPDH as loading control. The results shown are representative of three independent experiments (three replicates each). (B) Proliferation of transfected cells was measured using the Click iT EdU cell proliferation assay. The results are an average of three independent experiments (three replicates each). The error bars show standard error of mean. Student’s t-test was used to calculate statistical significance (*p<0.0005). Results shown are representative of three independent experiments.

The proliferation results were validated by over-expressing BNIP3 with a nuclear localization signal in HEK293 cells. These cells lack significant expression of BNIP3 under normal conditions and upon BNIP3 over-expression—in the nucleus fails to induce cell death. The cells were transfected with either an empty vector or a nuclear targeted BNIP3 vector. The western blot analysis of transfection can be seen in Fig 7A. Transfected cells were then analyzed for cell proliferation by Edu cell proliferation assay. Nuclear BNIP3 over-expressing cells showed reduced proliferation compared to vector alone cells (Fig 7B). Taken together, BNIP3 regulates cell proliferation in HEK 293 cells.

### Lack of BNIP3 expression is associated with increased cellularity in the mouse brain

**Fig 8:**
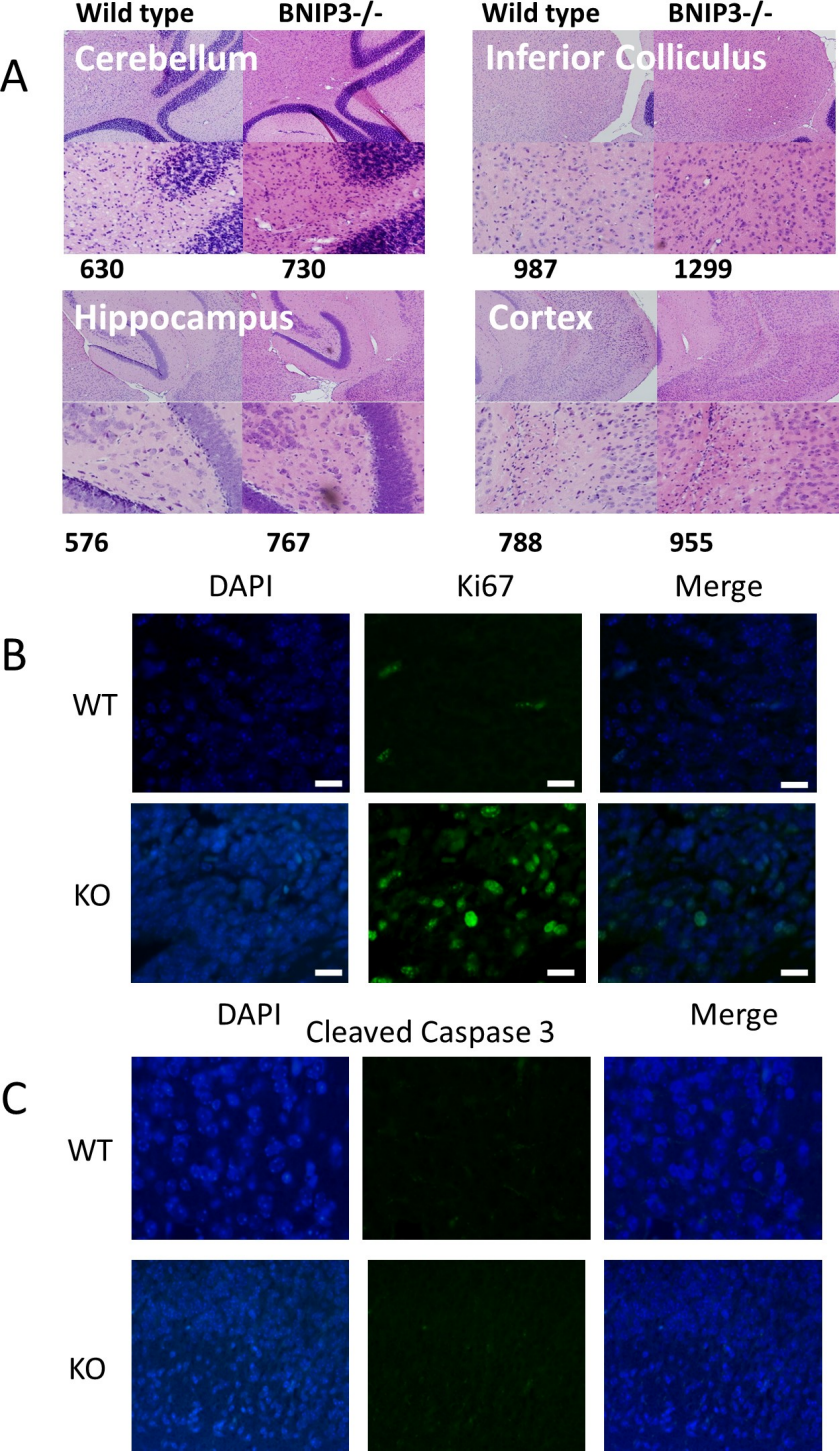
Morphology and cellularity changes in wild type and BNIP3 knockout brains. Whole brains from 8-week old wild type and BNIP3-/- mice were fixed by cardiac perfusion and overnight immersion in paraformaldehyde (n = 3 pairs of littermates from different heterozygous crosses). Brains were embedded in paraffin, sectioned and stained with hematoxylin and eosin as described in Materials & Methods. Images were captured at 10x and 40x magnification and cell counting was performed using Image Pro Plus 5.0 software. These representative images depict the morphology of the cerebellum, hippocampus, cortex and inferior colliculus, with cell counts for the 40x images indicating increased cellularity in BNIP3-/- brains. E18.5 embryos from B) wild type and C) BNIP3-/- mice were fixed as described in the Material and Methods section. The paraffin embedded brains were sliced and placed on slides and stained for DNA with DAPI and antibodies against Ki67 and leaved caspase 3. This images represent three independent experiments.

We next examined the gross brain morphology of unstressed adult mice. Wild-type and BNIP3 knockout littermates were sacrificed at 8 weeks of age (n = 3 pairs, each pair from a different litter). We found that BNIP3 knockout mice brains fail to exhibit any obvious gross abnormalities with normal expression of neurons and astrocytes (S3 Fig). Upon closer inspection, however, the BNIP3 knockout brains appeared more cellular compared to wild-type (Fig 8A). Although the difference was modest (average increase = 5%, 95% CI = -1% to 13%), it was consistently observed in multiple areas of the brain, including the cerebellum, hippocampus, cortex and inferior colliculus (Fig 8A).

We additionally examined brain morphology and cellularity in E18.5 embryos (n = 4 littermates, 2 wild -type and 2 BNIP3 knockout). Similar to adults, gross morphology appeared normal in BNIP3 knockout embryonic brains and moderately increased cellularity was observed (S4 Fig). The differential cellularity was statistically significant: E18.5 BNIP3 knockout brains were 11% more cellular compared to wild-type (95% confidence interval: 5.0–17.1%). Once again, increased cellularity was observed in multiple areas of the brain, including the: hippocampus, striatum, thalamus, cortex, stria terminalis, and paraventricular thalamic nucleus (Table 1). In agreement with these results, we found that during primary astrocyte isolation, cortices from BNIP3 knockout neonates contained more cells compared to wild-type; (2.68 x 105 cells/BNIP3-null cortex vs. 2.09 x 105 cells/wild-type cortex; n = 5 litters per genotype, representing 74 neonates in total). Finally, we conducted immunostaining of these brain sections with antibodies against Ki67 indicating increased proliferation and cleaved caspase 3 antibodies indicated apoptosis. We found that the BNIP3 knockout brain sections have increased Ki67 staining compared to wild type brain (Fig 8B) whereas cleaved caspase 3 staining remained unchanged (Fig 8C). These results suggest that in mice lacking BNIP3 expression brains have increased cell number consistent with increased cellular proliferation.

**Table 1:** Cellularity of wild-type and BNIP3 knockout E18.5 brains.

Region	Magnification	Wild type		BNIP# -/-		Ratio: null/WT
		Avg.	% SD	Avg.	% SD	
Hippocampus	20X	2410	11.0	2875	1.6	1.19
	40X	916	6.4	1284	5.9	1.4
Striatum	20X	3365	5.4	4057	9.6	1.21
	40X	1432	18.7	1577	1.0	1.10
Thalamus	20X	1954	20.0	2039	3.6	1.04
	40X	1221	12.0	1493	6.7	1.22
Somatosensory cortex	20X	2979	7.5	3798	4.8	1.28
	40X	1225	6.9	1275	4.6	1.04
Secondary auditory cortex	20X	3371	4.5	3286	5.3	0.97
	40X	1375	3.4	1287	5.9	0.94
Stria Terminalis	20X	2641	3.9	3057	N/A	1.16
	40X	646	2.8	688	4.7	1.07
Paraventricular thalamic nucleus	20X	2888	N/A	3144	5.4	1.09
	40X	1308	5.1	1336	2.2	1.02
**OVERALL TOTAL**						**1.11 (95% CL; 1.05–1.17)**

Whole brain sections from E18.5 embryos were prepared and analyzed ipp (n = 4 littermates, 2 wild-type and 2 BNIP3-/-). Average cell counts and percent standard deviation were calculated for 8 brain regions.

## Discussion

BNIP3 function has been extensively studied for its role in cell death and cell survival [11]. Our findings show that BNIP3 also plays an important role in regulating cell proliferation independent of cell death. This was demonstrated by showing that BNIP3 knockout cells had increased cell number, density and DNA synthesis in a variety of cell types. We also found increased cellularity in the brains of mice lacking BNIP3 expression indicating BNIP3 regulates cell number *in vivo*. Thus, we have described a new function for BNIP3.

Our findings showed increased MAPK activation in MEF cells lacking BNIP3. MAPK activation is associated with cellular proliferation following growth factor stimulation of many different cell types [12]. MAPK pathway activates as series of transcription factors and kinases that regulate cell cycle progression. Indeed, inhibition of MAPK has been shown as an attractive target for cancer therapy through blocking cell growth [13]. In a previous study, a rapamycin-sensitive growth advantage in BNIP3-knockdown tumor xenografts was shown, suggesting that BNIP3 inhibits cell growth by suppressing the mTOR pathway [14]. BNIP3 has been associated with the mTOR pathway through its association with Rheb [14]. MAPK and JNK pathway have been implicated in up-regulation of BNIP3 suggesting a negative feedback in MAPK regulation but the role BNIP3 plays in regulating signal transduction leading to cell proliferation is unknown [15] This will be the focus of future investigation.

Autophagy is an essential cellular function to maintain homeostasis in a cell [16]. Basal levels of autophagy regulate protein expression levels, metabolism and elimination of damaged organelles. Under cellular stress, autophagy levels increase to provide a survival advantage to cells especially under starvation conditions [17]. BNIP3 has been shown to target mitochondria for degradation through autophagy. This is mediated by its association with LC3 [18]. We found that inducible over-expression of BNIP3 increased autophagy associated with decreased cell growth but not cell death in HEK293 cells. Inhibition of autophagy has been shown to reduced tumor growth in tumor-bearing xenograft mice indicating it contributes to cell survival and growth of tumors [19]. We found no change in basal autophagy levels in cells lacking BNIP3 (data not shown). In contrast, under hypoxia BNIP3 is over expressed and induces autophagy. This contributes to cell death [20]. The role BNIP3-induced autophagy plays in cellular proliferation versus cell death might be context dependent and remains to be determined.

In astrocytes and glioma cells, BNIP3 is expressed in the nucleus [6]. Nuclear BNIP3 fails to induce cell death and following treatment with temozolomide protects cells from cell death. This was due to the repression of pro-apoptotic genes AIF and death receptor 5 (DR5) expression [6]. Other Bcl-2 family members have nuclear functions, such as Bcl-2 in regulating mitosis [21]. Even pro-apoptotic Bcl-2 family members were found in the nucleus [22]. Bid is involved in the cell cycle checkpoint response following DNA damage and BAX promotes cell proliferation by modulating CDKN1A expression [23]. It is conceivable that similar to other Bcl-2 family members, BNIP3 is regulating cell cycle progression through alterations in gene expression directly through nuclear localization or indirectly through alterations in signal transduction. We will further investigate these mechanisms of action in the future.

BNIP3 might play a role in brain development. A systematic evaluation of Bcl-2 family gene expression showed that BNIP3 is significantly upregulated during oligodendrocyte differentiation in an *in vitro* model [24]. In this study, differentiation of primary oligodendroglial progenitor cells was induced by withdrawal of trophic factors and gene expression was measured by real-time PCR. Levels of BNIP3 mRNA were increased more than four-fold during differentiation but not during proliferation. In our study, BNIP3 expression was detected in the neonatal cortex, hippocampus, habenula and thalamus, with the highest levels of expression occurring at postnatal day 6.5. This timing correlates with the previously described period of developmental apoptosis and reduced proliferation in the neonatal rat brain [25]. We have demonstrated that knockout of BNIP3 in the mouse brain results in increased cellularity and BNIP3 contributes to reduced proliferation without affecting cell death *in vitro*. This suggests that BNIP3 might play a role in brain development.

## Conclusion

Overall, we have identified a novel role for BNIP3 in regulating cell proliferation. These findings may ultimately have therapeutic implications in BNIP3-mediated proliferative pathways in ischemic injury or cancer.

## Supporting information

S1 Fig BNIP3 protein expression in mouse brain. Total cell lysates were generated from cryo-preserved brain tissue extracted from wild-type, heterozygous and BNIP3-null adult mice. Lysates were analyzed for BNIP3 expression by Western blot, with actin as a loading control; each lane represents a different mouse. Mice were sacrificed by cervical dislocation to minimize hypoxia at the time of death, and brain tissue was removed within 5 minutes.
(PDF)

S2 Fig MEF cells lacking BNIP3 have diminished levels of Cyclin D1 compared to MEF cells expressing BNIP3 after cell cycle synchronization. Similar to EdU assay, MEF cells expressing or lacking BNIP3 were serum starved overnight to synchronize cell cycle. In the morning, cells were supplied with 10% serum for 4 hours, and lysed for total protein. This time coincides with Edu analysis in Fig 2, where higher percentage of MEF cells lacking BNIP3 are in S-phase of cell cycle compared to cells expressing BNIP3. The lysates were western blotted for Cyclin D1. The blot was stripped and reprobed with GAPDH as loading control. The protein levels were quantified with ImageJ software, and are presented as a ratio of Cyclin D1 to GAPDH (normalized to the highest ratio). MEF cells lacking BNIP3 have lower levels of Cyclin D1 protein as would be expected since Cyclin D1 is degraded during S-phase of cell cycle.
(PDF)

S3 Fig Expression of neuronal and astrocyte markers in wild type and BNIP3-/- mouse brain. Wild-type and BNIP3-/- mice were sacrificed at 8–32 weeks of age and brains were cryopreserved as described in Materials and Methods. (A) Detection of the astrocyte marker GFAP (glial fibrillary acidic protein) in adult (8 week) mouse brain by immunofluorescence. (B) Detection of GFAP and the neuronal marker NF-L (68kDa light neurofilament subunit) in cultured astrocytes (Ast.) and adult (8–32 week) mouse brains. To control for loading, the Bradford protein assay was performed on all lysates and an equal amount of total protein was loaded in each lane.
(PDF)

S4 Fig Morphology and cellularity of E18.5 wild-type and BNIP3 knockout mice. E18.5 embryos were obtained from a single heterozygous cross. Brains were fixed by overnight immersion in paraformaldehyde, followed by paraffin embedding, horizontal sectioning and staining with hematoxylin and eosin. Images captured at 40x magnification revealed no significant difference in general morphology; representative images are shown in (A). Images captured at 20x and 40x magnification were analyzed with Image Pro Plus 5.0 to determine cellularity; representative images with cell counts are shown in (B). These images correspond to region “C” in panel (C), which depicts the 8 regions analyzed for cellularity in each brain. A = hippocampus, B = striatum, C = thalamus, D = somatosensory cortex, E = hippocampus, F = secondary auditory cortex, G = stria terminalis, H = paraventricular thalamic nucleus.
(PDF)

S1 File Data for figures. (XLSX)
