# Supplementary material for: The BH3 only Bcl-2 family member BNIP3 regulates cellular proliferation
Source: PLoS One. 2018 Oct 11;13(10):e0204792. doi: 10.1371/journal.pone.0204792 (PMC6181300; doi:10.1371/journal.pone.0204792)
Supplement: S1 Fig — Total cell lysates were generated from cryo-preserved brain tissue extracted from wild-type, heterozygous and BNIP3-null adult mice. Lysates were analyzed for BNIP3 expression by Western blot, with actin as a loading control; each lane represents a different mouse. Mice were sacrificed by cervical dislocation to minimize hypoxia at the time of death, and brain tissue was removed within 5 minutes. (PDF) [file pone.0204792.s001.pdf]

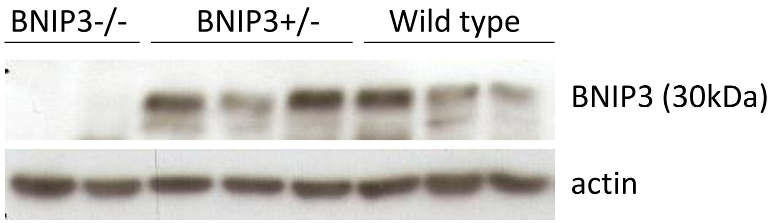

S1 Fig BNIP3 protein expression in mouse brain.
Total cell lysates were generated from cryo-preserved brain tissue extracted from wild-type, heterozygous and BNIP3-null adult mice. Lysates were analyzed for BNIP3 expression by Western blot, with actin as a loading control; each lane represents a different mouse. Mice were sacrificed by cervical dislocation to minimize hypoxia at the time of death, and brain tissue was removed within 5 minutes.
