# Supplementary material for: The BH3 only Bcl-2 family member BNIP3 regulates cellular proliferation
Source: PLoS One. 2018 Oct 11;13(10):e0204792. doi: 10.1371/journal.pone.0204792 (PMC6181300; doi:10.1371/journal.pone.0204792)
Supplement: S2 Fig — Similar to EdU assay, MEF cells expressing or lacking BNIP3 were serum starved overnight to synchronize cell cycle. In the morning, cells were supplied with 10% serum for 4 hours, and lysed for total protein. This time coincides with Edu analysis in Fig 2, where higher percentage of MEF cells lacking BNIP3 are in S-phase of cell cycle compared to cells expressing BNIP3. The lysates were western blotted for Cyclin D1. The blot was stripped and reprobed with GAPDH as loading control. The protein levels were quantified with ImageJ software, and are presented as a ratio of Cyclin D1 to GAPDH (normalized to the highest ratio). MEF cells lacking BNIP3 have lower levels of Cyclin D1 protein as would be expected since Cyclin D1 is degraded during S-phase of cell cycle. (PDF) [file pone.0204792.s002.pdf]

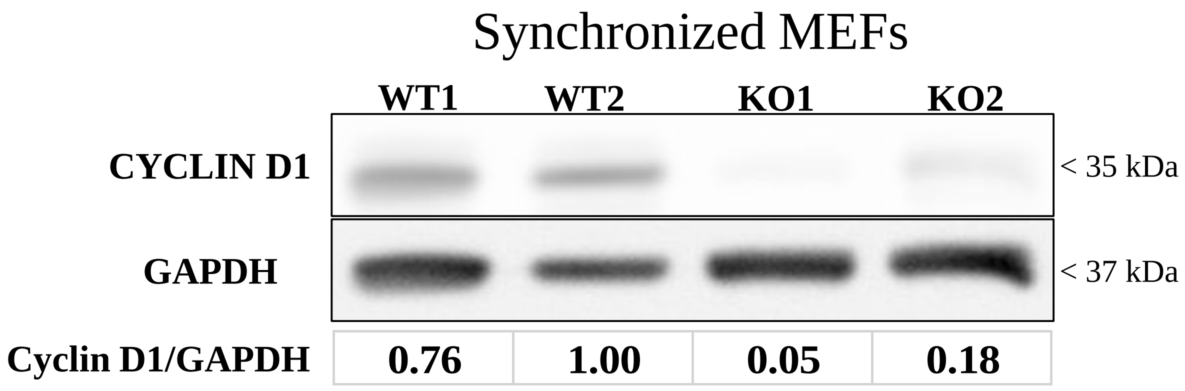

S2 Fig: MEF cells lacking BNIP3 have diminished levels of Cyclin D1 compared to MEF cells expressing BNIP3 after cell cycle synchronization.

Similar to EdU assay, MEF cells expressing or lacking BNIP3 were serum starved overnight to synchronize cell cycle. In the morning, cells were supplied with 10% serum for 4 hours, and lysed for total protein. This time coincides with Edu analysis in figure 2, where higher percentage of MEF cells lacking BNIP3 are in S-phase of cell cycle compared to cells expressing BNIP3. The lysates were western blotted for Cyclin D1. The blot was stripped and reprobed with GAPDH as loading control. The protein levels were quantified with ImageJ software, and are presented as a ratio of Cyclin D1 to GAPDH (normalized to the highest ratio). MEF cells lacking BNIP3 have lower levels of Cyclin D1 protein as would be expected since Cyclin D1 is degraded during S-phase of cell cycle.
