# Supplementary material for: The BH3 only Bcl-2 family member BNIP3 regulates cellular proliferation
Source: PLoS One. 2018 Oct 11;13(10):e0204792. doi: 10.1371/journal.pone.0204792 (PMC6181300; doi:10.1371/journal.pone.0204792)
Supplement: S3 Fig — Wild-type and BNIP3-/- mice were sacrificed at 8–32 weeks of age and brains were cryopreserved as described in Materials and Methods. (A) Detection of the astrocyte marker GFAP (glial fibrillary acidic protein) in adult (8 week) mouse brain by immunofluorescence. (B) Detection of GFAP and the neuronal marker NF-L (68kDa light neurofilament subunit) in cultured astrocytes (Ast.) and adult (8–32 week) mouse brains. To control for loading, the Bradford protein assay was performed on all lysates and an equal amount of total protein was loaded in each lane. (PDF) [file pone.0204792.s003.pdf]

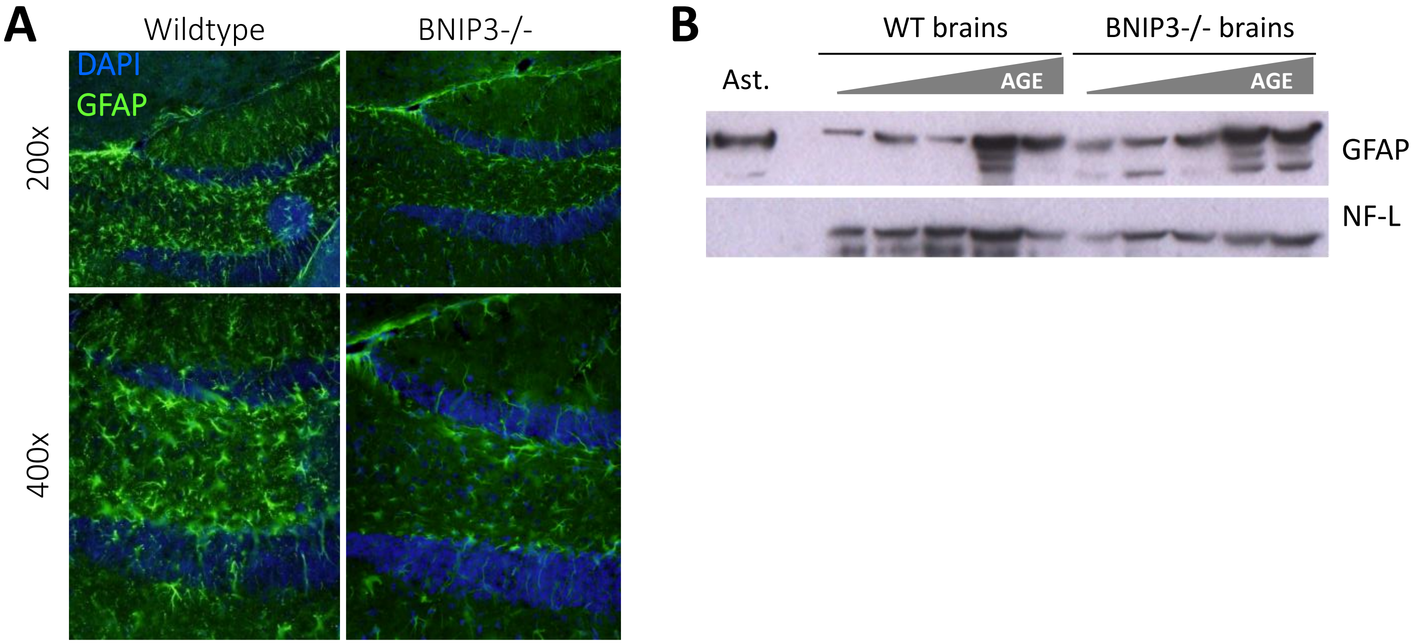

S3 Fig Expression of neuronal and astrocyte markers in wild type and BNIP3-/- mouse brain. Wild-type and BNIP3-/- mice were sacrificed at 8 – 32 weeks of age and brains were cryopreserved as described in Materials and Methods. (A) Detection of the astrocyte marker GFAP (glial fibrillary acidic protein) in adult (8 week) mouse brain by immunofluorescence. (B) Detection of GFAP and the neuronal marker NF-L (68kDa light neurofilament subunit) in cultured astrocytes (Ast.) and adult (8 - 32 week) mouse brains. To control for loading, the Bradford protein assay was performed on all lysates and an equal amount of total protein was loaded in each lane.
