# Supplementary material for: The BH3 only Bcl-2 family member BNIP3 regulates cellular proliferation
Source: PLoS One. 2018 Oct 11;13(10):e0204792. doi: 10.1371/journal.pone.0204792 (PMC6181300; doi:10.1371/journal.pone.0204792)
Supplement: S4 Fig — E18.5 embryos were obtained from a single heterozygous cross. Brains were fixed by overnight immersion in paraformaldehyde, followed by paraffin embedding, horizontal sectioning and staining with hematoxylin and eosin. Images captured at 40x magnification revealed no significant difference in general morphology; representative images are shown in (A). Images captured at 20x and 40x magnification were analyzed with Image Pro Plus 5.0 to determine cellularity; representative images with cell counts are shown in (B). These images correspond to region “C” in panel (C), which depicts the 8 regions analyzed for cellularity in each brain. A = hippocampus, B = striatum, C = thalamus, D = somatosensory cortex, E = hippocampus, F = secondary auditory cortex, G = stria terminalis, H = paraventricular thalamic nucleus. (PDF) [file pone.0204792.s004.pdf]

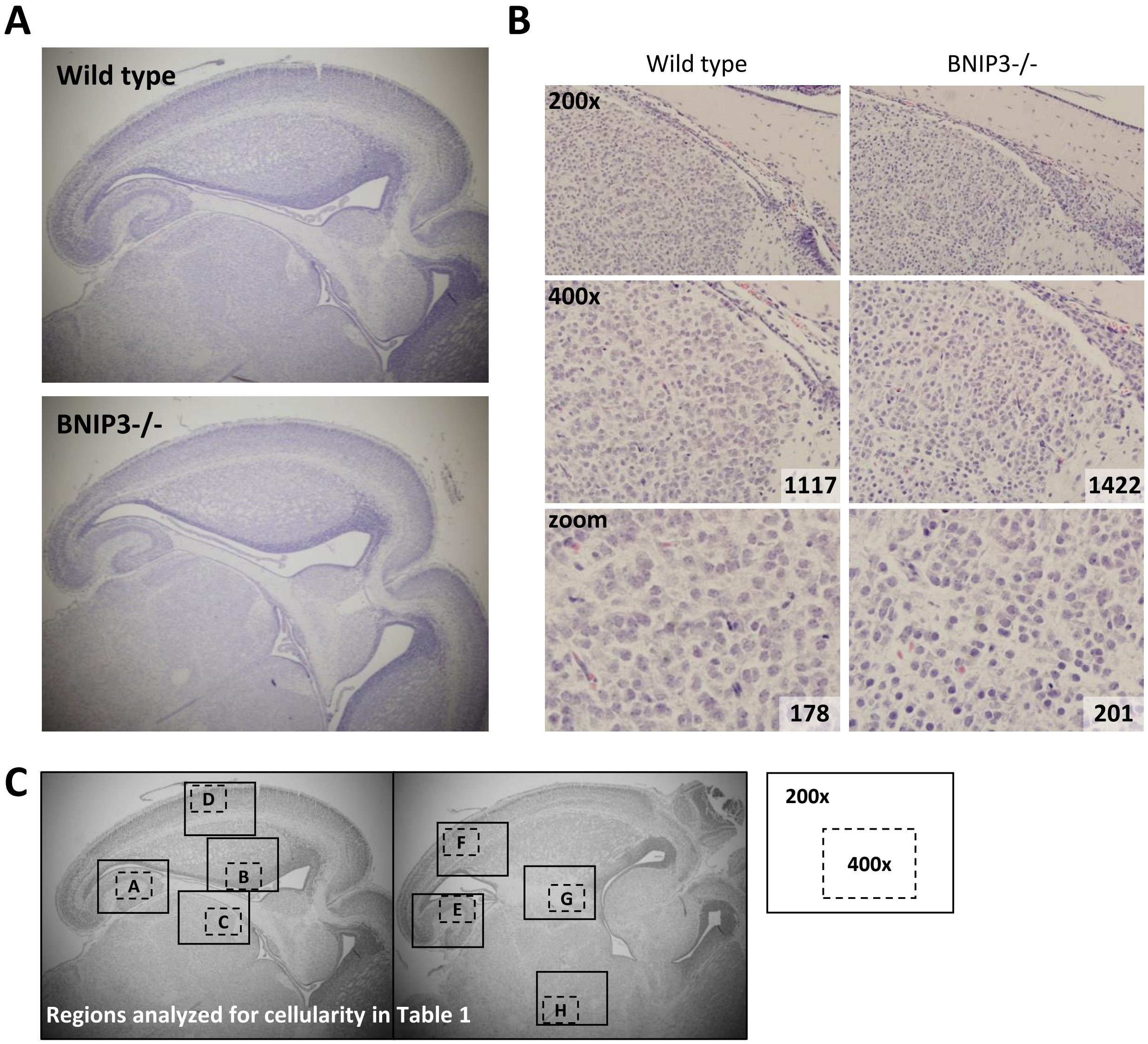

S4 Fig Morphology and cellularity of E18.5 wild-type and BNIP3 knockout mice. E18.5 embryos were obtained from a single heterozygous cross. Brains were fixed by overnight immersion in paraformaldehyde, followed by paraffin embedding, horizontal sectioning and staining with hematoxylin and eosin. Images captured at 40x magnification revealed no significant difference in general morphology; representative images are shown in (A). Images captured at 20x and 40x magnification were analyzed with Image Pro Plus 5.0 to determine cellularity; representative images with cell counts are shown in (B). These images correspond to region “C” in panel (C), which depicts the 8 regions analyzed for cellularity in each brain. A = hippocampus, B = striatum, C = thalamus, D = somatosensory cortex, E = hippocampus, F = secondary auditory cortex, G = stria terminalis, H = paraventricular thalamic nucleus.
